# Dual Inhibition of AKT and MEK Pathways Potentiates the Anti-Cancer Effect of Gefitinib in Triple-Negative Breast Cancer Cells

**DOI:** 10.3390/cancers13061205

**Published:** 2021-03-10

**Authors:** Kyu Sic You, Yong Weon Yi, Jeonghee Cho, Yeon-Sun Seong

**Affiliations:** 1 Graduate School of Convergence Medical Science, Dankook University, Cheonan 31116, Korea; kisuhezu@gmail.com; 2 Department of Biochemistry, College of Medicine, Dankook University, Cheonan 31116, Korea; 3 Department of Nanobiomedical Science, Dankook University, Cheonan 31116, Korea; dragon101@gmail.com

**Keywords:** AKT, anti-cancer, combination, EGFR resistance, MEK, protein kinase inhibitor (PKI), synergism, triple-negative breast cancer (TNBC)

## Abstract

**Simple Summary:**

Triple-negative breast cancer (TNBC) is an intractable subset of breast cancer without an efficient therapeutic strategy due to the lack of tractable targets, such as the estrogen receptor, progesterone receptor, and human epidermal growth factor receptor 2. Activation and/or amplification of epidermal growth factor receptor (EGFR), phosphoinositide 3-kinase (PI3K)/v-akt murine thymoma viral oncogene homolog (AKT), and MAPK/ERK kinase (MEK)/ extracellular signal-regulated kinase (ERK) pathways is known in TNBC; however, interventions for these targets have not been successful to date. To explore a combinatorial strategy with existing clinical/preclinical protein kinase inhibitors (PKIs) in TNBC cells, we performed a series of cytotoxicity (cell viability) screenings with various PKIs in the presence figure of an EGFR inhibitor, gefitinib. The dual inhibition of AKT and MEK with gefitinib reduced the proliferation and colony formation of TNBC cells by inducing apoptosis. Our finding suggests a new approach for treating TNBC with a multiplex combination of PKIs.

**Abstract:**

There is an unmet medical need for the development of new targeted therapeutic strategies for triple-negative breast cancer (TNBC). With drug combination screenings, we found that the triple combination of the protein kinase inhibitors (PKIs) of the epidermal growth factor receptor (EGFR), v-akt murine thymoma viral oncogene homolog (AKT), and MAPK/ERK kinase (MEK) is effective in inducing apoptosis in TNBC cells. A set of PKIs were first screened in combination with gefitinib in the TNBC cell line, MDA-MB-231. The AKT inhibitor, AT7867, was identified and further analyzed in two mesenchymal stem-like (MSL) subtype TNBC cells, MDA-MB-231 and HS578T. A combination of gefitinib and AT7867 reduced the proliferation and long-term survival of MSL TNBC cells. However, gefitinib and AT7867 induced the activation of the rat sarcoma (RAS)/ v-raf-1 murine leukemia viral oncogene homolog (RAF)/MEK/ extracellular signal-regulated kinase (ERK) pathway. To inhibit this pathway, MEK/ERK inhibitors were further screened in MDA-MB-231 cells in the presence of gefitinib and AT7867. As a result, we identified that the MEK inhibitor, PD-0325901, further enhanced the anti-proliferative and anti-clonogenic effects of gefitinib and AT7867 by inducing apoptosis. Our results suggest that the dual inhibition of the AKT and MEK pathways is a novel potential therapeutic strategy for targeting EGFR in TNBC cells.

## 1. Introduction

Triple-negative breast cancer (TNBC) is defined clinically as being negative for both the estrogen receptor (ER) and progesterone receptor (PR) and having no amplification of human epidermal growth factor receptor 2 (HER2) [1,2]. Therefore, the therapeutic options that feature drugs targeting these proteins are not available for TNBC patients. TNBC accounts for approximately 15–20% of breast cancers and is known as the most aggressive breast cancer [1,2,3,4,5,6]. Although TNBCs respond well to conventional adjuvant chemotherapies, including taxanes, anthracyclines, capecitabines, and/or platins, resistance to these agents and distant metastasis diminish overall prognosis [7,8,9,10]. Experimental therapeutics against specific targets, such as acetylated signal transducer and activator of transcription 3 (STAT3) and mutant p53 in TNBC cells, have been developed [11,12,13]. Up to date, four targeted therapies have been approved by the US Food and Drug Administration (FDA). These therapies include the poly [adenosine diphosphate (ADP)-ribose] polymerase 1 (PARP1) inhibitors, olaparib and talazoparib; the programmed cell death ligand 1 (PD-L) inhibitor, atezolizumab; and antibody drug conjugates (ADCs), sacituzumab govitecan [2]. However, most treatments exhibit limited durable clinical responses [2].

The epidermal growth factor receptor (EGFR) is a member of the human epidermal growth factor receptor (HER) family, which consists of four receptor tyrosine kinases that are important for the proliferation and survival of normal cells [14,15,16]. EGFR is activated via epidermal growth factor (EGF) binding, which then promotes intracellular signaling pathways, such as rat sarcoma (RAS)/v-raf-1 murine leukemia viral oncogene homolog (RAF)/MAPK/ERK kinase (MEK)/extracellular signal-regulated kinase (ERK), phosphoinositide 3-kinase (PI3K)/v-akt murine thymoma viral oncogene homolog (AKT)/mammalian target of rapamycin (mTOR), and v-src avian sarcoma (Schmidt-Ruppin A-2) viral oncogene homolog (SRC)/signal transducer and activator of transcription (STAT) [17,18,19]. Activation of EGFR, that caused by *EGFR* gene amplification or mutations, or protein overexpression, or point mutations has been reported in many cancer types. EGFR is a well-established therapeutic target; many small-molecule kinase inhibitors and monoclonal antibodies have been approved for treating several human cancers by the US FDA [15,16]. High EGFR expression has been reported in >50% of TNBC, which is associated with a poor prognosis [1,3,14,15,20]. Lehmann et al. have classified TNBC into six subtypes and shown that two of them have the active EGFR pathway: basal-like 2 (BL2) and mesenchymal stem-like (MSL) subtypes [5]. However, TNBC has displayed intrinsic resistance to anti-EGFR therapeutics [3,20]. One possible explanation is that most TNBCs are not solely dependent on the EGFR pathway for their survival because of rare EGFR-activating mutations [3]. Most anti-EGFR therapeutics are effective in cancers that have activated mutations in EGFR.

Combining existing therapeutics is a promising way to treat intractable cancers, such as pancreatic cancer or TNBC [2,21,22,23,24,25,26,27,28,29,30,31,32,33,34]. For example, blocking the PI3K/AKT pathway [25], MET [30], or mammalian target of rapamycin complex 1 (mTORC1) [33] sensitized TNBC cells to EGFR inhibitors (EGFRis). A combination of EGFRi, gefitinib, or erlotinib with PI3K/AKT inhibitors resulted in the synergism of an anti-proliferative effect in the cell lines of the BL subtype [25]. However, these combinations have no synergism in the MSL subtype cell lines. Additionally, we determined that co-treatment with the MET inhibitor (METi), SU11274, and EGFRis has a synthetic lethality in MSL TNBC cells though the downregulation of ribosomal protein S6 (RPS6) [30]. Additionally, inhibiting the mTORC1 pathway via the AKT inhibitor, MK2206, or blocking the regulatory-associated protein of mTOR (RPTOR) with small interfering RNA (siRNA) potentiated gefitinib toxicity in TNBC cells [33]. Recently, more efficacious treatments for TNBC have been suggested that use a triple combination of drugs targeting multiple pathways simultaneously, such as redox homeostasis, DNA synthesis, DNA damage, histone deacetylase, and multiple protein kinases [35,36,37]. A drug combination discovery involving 33 FDA-approved PKIs revealed that the triple combination of dasatinib, afatinib (BIBW-2992), and trametinib (GSK1120212) was anti-proliferative in TNBC cells by inhibiting SRC, HER2/EGFR, and MEK [37,38,39,40].

In this paper, we showed that the dual blocking of the AKT and MEK pathways sensitized TNBC cells to the EGFRi, gefitinib. A set of small-molecule PKIs were screened in combination with gefitinib for the MSL subtype cell, MDA-MB-231. An AKT inhibitor (AKTi), AT7867, was identified as the most potent inhibitor, which we further analyzed using two MSL subtype TNBC cells, MDA-MB-231 and HS578T. A combination of gefitinib and AT7867 reduced the proliferation and long-term survival of MSL TNBC cells. However, gefitinib and AT7867 (hereafter referred to as Gefi+AT7867) induced the activation of the MEK/ERK pathway. Blocking this pathway with the MEK inhibitor (MEKi), PD-0325901, further enhanced the anti-cancer effect of Gefi+AT7867. Our results suggest that the dual inhibition of the AKT and MEK pathways is a potential therapeutic strategy for targeting EGFR in TNBC cells.

## 2. Materials and Methods

### 2.1. Cell Culture and Reagents

The MDA-MB-231 and HS578T cell lines were obtained from the American Type Culture Collection (ATCC; Manassas, VA, USA). Cells were cultured in Dulbecco’s modified Eagle’s medium (DMEM; HyClone, Logan, UT, USA) supplemented with 10% fetal bovine serum (FBS; Corning, Manassas, VA, USA) and 100 units/mL penicillin/streptomycin (Thermo Fisher Scientific, Waltham, MA, USA). The viability of cultured cells were monitored by the Trypan Blue dye exclusion method using an automated cell counter, as described previously [41]. The PKIs used in this study were reported in Table 1.

**Table 1:** Protein kinase inhibitors (PKIs) used in 3-(4,5-dimethylthiazol-2-yl)-2,5-diphenyltetrazolium bromide (MTT) screening.

PKI	Other Name	Known Targets (IC_50_ Value in nM)	Source	Ref
A-769662		AMPK (800; EC_50_)	LC Laboratories (Woburn, MA, USA)	[42]
AT7867		AKT2 (17), PKA (20), AKT1 (32), AKT3 (47), p70S6K (85)	Selleck Chemicals (Houston, TX, USA)	[43]
AT9283		JAK3 (1.1), JAK2 (1.2), AURKA (~3.0), AURKB (~3.0), ABL1^T315I^ (4)		[44]
AZD1152-HQPA	Barasertib, AZD2811	AURKB (0.37)		[45]
AZD1480		JAK2 (0.26)		[46]
BI 2536		PLK1 (0.83), PLK2 (3.5)		[47,48]
BIX 02189		MEK5 (1.5)		[49]
BML-275	Dorsomorphin, Compound C	AMPK (109; Ki)	Tocris Bioscience (Bristol, UK)	[50]
Bosutinib	SKI-606	ABL (1), SRC (1.2)	LC Laboratories	[51,52]
Chelerythrine		PKC (660)		[53]
CHIR-99021	CT99021	GSK3β (6.7), GSK3α (10)	Selleck Chemicals	[54]
CI-1040	PD184352	MEK1 (17), MEK2 (17)		[55]
CP690550	Tofacitinib	JAK3 (1), JAK2 (20)	LC Laboratories	[56]
CYC116		AURKA (8; Ki), AURKB (9; Ki), VEGFR2 (44; Ki), FLT3 (44; Ki)	Selleck Chemicals	[57]
Danusertib	PHA-739358	AURKA (13), ABL (25), RET (31), TRKA (31), FGFR1 (47), AURKC (61), AURKB (79)		[58]
Enzastaurin	LY317615	PKCβ (6), PKCα (39), PKCγ (83), PKCε (110)		[59]
Fasudil	HA-1077	ROCK2 (330)	LC Laboratories	[60]
FR 180204		ERK2 (140), ERK1 (310)	Tocris Bioscience	[61]
GDC-0879	AR-00341677	BRAF (0.13)	Selleck Chemicals	[62]
GW 843682X		PLK1 (2.2), PLK3 (9.1)	Tocris Bioscience	[63]
I3M		GSK3β (190)	Calbiochem (San Diego, CA, USA)	[64]
IKK 16	IKK Inhibitor VII	IKK2 (40), IKK complex (70), IKK1 (200)	Tocris Bioscience	[65]
Imatinib	STI571, CGP057148B, Gleevec	PDGFR (100), c-KIT (100), v-ABL (600)	LC Laboratories	[66]
INCB018424	Ruxolitinib	JAK2 (2.8), JAK1 (3.3)	Selleck Chemicals	[67]
JNJ-7706621		CDK2/Cyclin E (3), CDK2/Cyclin A (4), CDK1/Cyclin B (9), AURKA (11), AURKB (15)		[68]
KU-55933		ATM (12.9)		[69]
LY2228820	Ralimetinib	P38α (7)		[70]
MLN8237	Alisertib	AURKA (1.2)		[71]
Nilotinib	AMN-107	Bcr-Abl (<30)	LC Laboratories	[72]
NSC 109555		CHK2 (200)	Tocris Bioscience	[73]
NU 7441	KU-57788	DNA-PK (14)		[74]
PD-0325901	Mirdametinib	MEK (0.33)	Selleck Chemicals	[75]
PD407824		CHK1 (47), WEE1 (97)	Tocris Bioscience	[76]
PF-4708671		p70S6K1 (160)	Selleck Chemicals	[77]
PF 573228		FAK (4)	Tocris Bioscience	[78]
PKC412	Midostaurin, CGP 41251	PKCα (22), PKCγ (24), PKCβ1 (30), PKCβ2 (31), PPK (38)	LC Laboratories	[79]
PLX-4032	Vemurafenib	SRMS (18), ACK (19), BRAF (31), C-RAF (48), MAP4K5 (51)	Selleck Chemicals	[80]
PLX-4720		BRAF^V600E^ (13), C-RAF1^Y340D/Y341D^ (6.7)		[81]
Ro-31-8220	Bisindolylmaleimide IX	PKCα (5), PKCβ2 (14), PKCβ1 (24), PKCε (24), PKCγ (27)	Calbiochem	[82]
Roscovitine	Seliciclib, CYC202	CDK5/P35 (160)	LC Laboratories	[83]
SB216763		GSK3α (34.3), GSK3β (~34.3)	Selleck Chemicals	[84]
SB 218078		CHK1 (15)	Tocris Bioscience	[85]
SNS-032	BMS-387032	CDK9/Cyclin T (4)	Selleck Chemicals	[86]
SNS-314		AURKC (3), AURKA (9), AURKB (31)		[87]
SP600125	NSC75890	JNK1 (40), JNK2 (40), AURKA (60), TRKA (70)		[88]
TBCA		CK2 (110)	Millipore (Burlington, MA, USA)	[89]
TCS 2312		CHK1 (60; EC_50_)	Tocris Bioscience	[90]
TC PIM-1-1	SC 204330	PIM (50)		[91]
TCS PIM-1-4a	SMI-4a	PIM1 (17)		[92]
Tozasertib	VX-680, MK-0457	AURKA (0.6; Ki), AURKC (4.6; Ki), AURKB (18; Ki), FLT3 (30; Ki), BCL-ABL (30; Ki)	LC Laboratories	[93,94]
TPCA-1	GW683965	IKK2 (17.9)	Tocris Bioscience	[95]
U0126		MEK2 (60), MEK1 (70)	Promega (Madison, WI, USA)	[96]
VX-702		P38α (4–20)	Selleck Chemicals	[97]
Y-27632		ROCK1 (140; Ki); ROCK2 (300; Ki)		[98,99]
ZM-447439		AURKA (110), AURKB (130)		[100]

### 2.2. PKI Screening

A synthetic lethality screening of PKIs was performed, as described previously [30,33]. Briefly, MDA-MB-231 cells were plated at 1000 cells/well in 96-well plates and incubated for 24 h. The cells were treated with increasing concentrations of gefitinib and PKIs in duplicate in a 6 × 5 matrix that included a dimethyl sulfoxide (DMSO) vehicle control. The viability of cells was determined at 72 h after treatment with 4 mg/mL of 3-(4,5-dimethylthiazol-2-yl)-2,5-diphenyltetrazolium bromide (MTT), as described previously [11,25,101]. The classification index (CI) was calculated to determine synergism of the combination, as described previously [30,33]: CI = (viability with gefitinib) × (viability with PKI) / (viability with the gefitinib and PKI combo). CI > 1, supra-additivity; CI = 1, additivity; and CI < 1, sub-additivity. The numbers of combination points with CI > 1.3 were assigned as quantitative indices of synergism.

### 2.3. Clonogenic Survival Assay

Cells were seeded and cultured in 6-well plates, as described previously [33]. Cells in each well were treated with indicated drugs, and the colonies were stained with crystal violet solution as previously described [30]. After washing the colonies with distilled water, they were imaged with an image scanner.

### 2.4. Western Blot Analysis

For the treatment of PKI, the cells were plated at 2 × 10^5^ cells/60-mm dish and incubated for approximately 24 h. Next, cells were treated with PKIs for 2 h or 24 h in normal growth media supplemented with FBS. Following treatment, the cells were lysed with a radioimmunoprecipitation assay (RIPA) buffer containing a protease and phosphatase inhibitor cocktail (ThermoFisher Scientific, Waltham, MA, USA). The protein concentration was measured via bicinchoninic acid (BCA) assay (Thermo Fisher Scientific, Waltham, MA, USA). The antibodies used in this study as follows: mTOR, p-mTOR (S2448), ERK1/2, p-ERK1/2 (T202/Y204), RPS6, p-RPS6 (S235/236), AKT, p-AKT (S473), glycogen synthase kinase-3 beta (GSK3β), p-GSK3β (S9), p-p90 ribosomal S6 kinase (RSK) (S380), p90RSK, p-STAT3 (T705), STAT3, cleaved caspase-3, and peroxidase-conjugated secondary antibodies (anti-rabbit IgG and anti-mouse IgG) from Cell Signaling Technology (Denver, MA, USA); XIAP from BD Sciences (San Jose, CA, USA); β-actin from Bethyl Laboratories (Montgomery, TX, USA); and β-tubulin from Sigma-Aldrich (St. Louis, MI, USA).

### 2.5. Cell Cycle Analysis

Cells were plated at 1 × 10^5^ cells in 60-mm dishes one day before treatment. Following the drug treatment, both adherent and floating cells were collected and fixed using 70% ethanol, as described previously [33]. The nuclei were stained with propidium iodide (PI) (Sigma-Aldrich; St. Louis, MI, USA), and the DNA contents were determined using a FACSCalibur Flow Cytometer (BD Sciences; Franklin Lakes, NJ, USA). The data were analyzed using CellQuest Pro (BD Sciences; Franklin Lakes, NJ, USA) and the ModFit LT^TM^ program (Verity Software House; Topsham, ME, USA).

### 2.6. Antibody Array

An antibody array analysis was performed by Fullmoon Biosystems (Sunnyvale, CA, USA) through ebiogen (Seoul, Korea). In brief, the protein extracts were purified by a gel matrix column. The protein extract quality was accessed via BCA protein assay with wavelength scanning using a spectrophotometer Model 680 (Bio-Rad Laboratories, Hercules, CA, USA). Fifty micrograms of each protein extract were used to label and probe the antibody array. Fluorescence signals were captured using the GenePix 4000B Microarray Scanner (Molecular Devices; San Jose, CA, USA). The signal intensity of each spot was normalized using global normalization.

### 2.7. Statistical Analysis

All experiments were performed at least three times in triplicate. Representative data are presented as means ± standard deviation (SD). The differences between groups were determined by the One-way analysis of variance (ANOVA) with a *post-hoc* Tukey’s honest significance difference (HSD) test. Data were considered statistically significant when *p* < 0.05.

## 3. Results

### 3.1. Identification of AT7867 as a Potentiator of Gefitinib in MDA-MB-231 Cells

**Figure 1:**
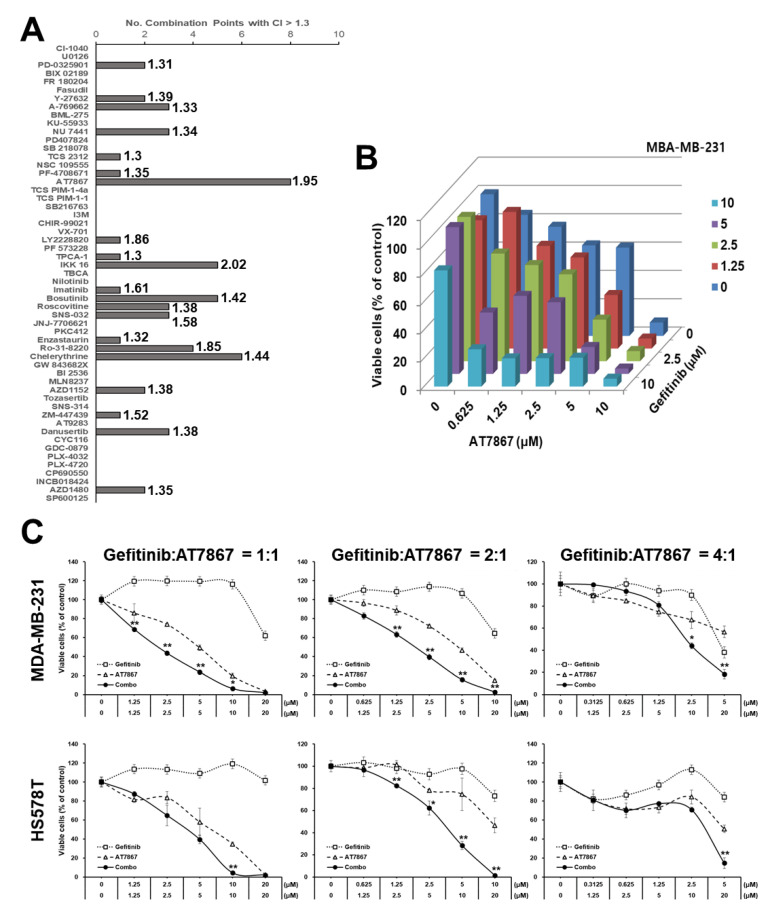
Treatment with AT7867 induced triple-negative breast cancer (TNBC) cells’ sensitivity to gefitinib. (**A**) PKI + gefitinib screening results in MDA-MB-231 cells. The numbers of combination points with CI > 1.3 was indexed (see the Materials and Methods). The numbers on the right of each bar are the mean CI values of indicated drugs. (**B**) MTT screening results for AT7867 with gefitinib. MDA-MB-231 cells were treated with an increasing concentrations of AT7867 and gefitinib for 72 hr. The viable cells was measured by MTT assay. (**C**) The combination effect of gefitinib with AT7867 in two MSL TNBC cells. Data represent mean ± SD from at least three independent experiments performed in triplicate. * *p* < 0.05 and ** *p* < 0.01.

Previously, we identified a series of PKIs that potentiate TNBC cells’ sensitivity to EGFRis [25,30,33]. Our studies also revealed the differential susceptibility of EGFRis and PKI combinations in subtypes of TNBC cells in vitro; MSL subtype cells are more resistant to PI3K/AKTi and EGFRi combinations than BL subtype cells [25]. This may be due to alternative survival pathways [102,103,104,105,106,107]. To further identify synergistic anti-cancer effects in MSL TNBC cells that overcome EGFRi resistance, we performed a synthetic lethality MTT screening with a new set of PKIs in combination with the EGFRi, gefitinib, in MDA-MB-231 cells. Among 55 PKIs tested, AT7867 was identified as the most promising PKI with a CI larger than 1.3, in 8 combination points from the 6 × 5 combination matrix (Figure 1A,B). In addition, its mean CI was relatively high (1.95; Figure 1A). AT7867 is an adenosine triphosphate (ATP)-competitive small-molecule PKI of AKT and its downstream kinase, p70 S6 kinase (p70S6K), as well as of protein kinase A (PKA) [43]. The synergistic effect of AT7867 and gefitinib was observed in a series of concentrations at different combination ratios in MDA-MB-231 cells (Figure 1C). The synthetic lethal effect of AT7867 with gefitinib was also confirmed in an additional MSL cell, HS578T (Figure 1C).

### 3.2. Induction of Tetraploid Cells by AT7867

**Figure 2:**
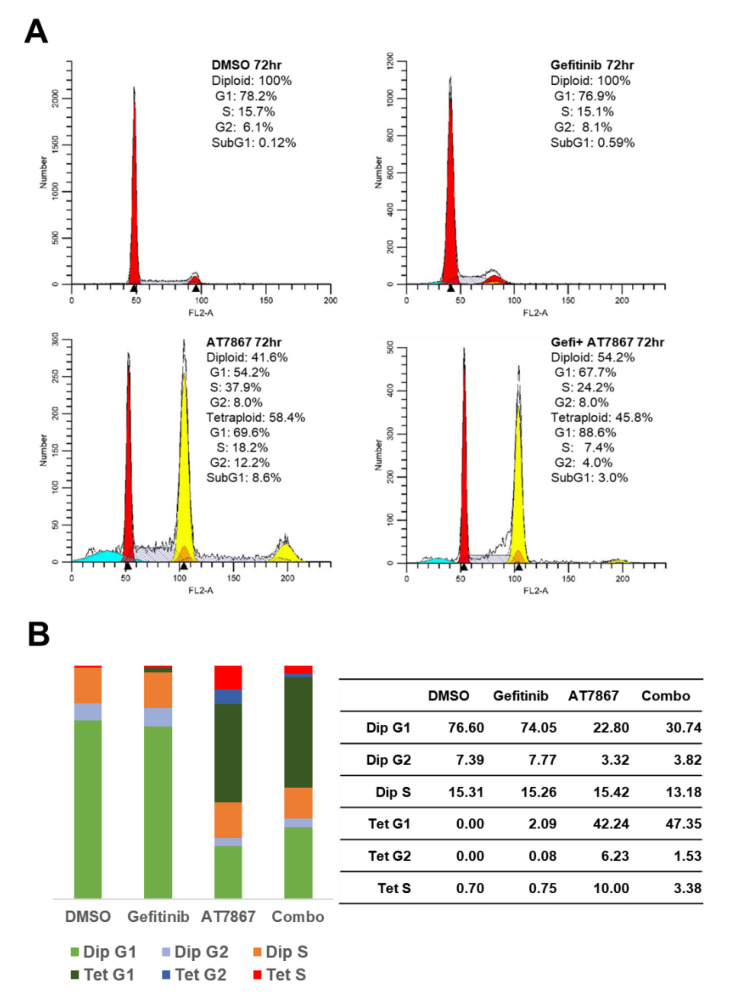
A combination of AT7867 and gefitinib-induced tetraploid gap 1 (G1) arrest in MDA-MB-231 cells. (**A**) Representative histograms of cell cycle analysis in MDA-MB-231 cells in the presence of each drug or gefitinib and AT7867 (Gefi+AT7867). (**B**) Relative distribution of cell cycle phases.

The effect of the Gefi+AT7867 treatment for 72 h on cell cycle distribution was determined in MDA-MB-231 cells. The cell cycle distribution was determined by flow cytometric analysis (Figure 2). Gefitinib treatment alone did not alter cell cycle progression in MDA-MD-231 cells; however, AT7867 induced an increase in the population of tetraploid cells (Figure 2A). Gefi+AT7867 further induced an increase of the gap 1 (G1) population with a concomitant reduction of the synthesis (S) and gap 2 (G2) populations in tetraploid cells (Figure 2B). Failures in spindle assembly, chromosome segregation, or cytokinesis followed by mitosis cause tetraploidy [108].

### 3.3. Gefitinib and AT7867 Reduced TNBC Cell Survival

**Figure 3:**
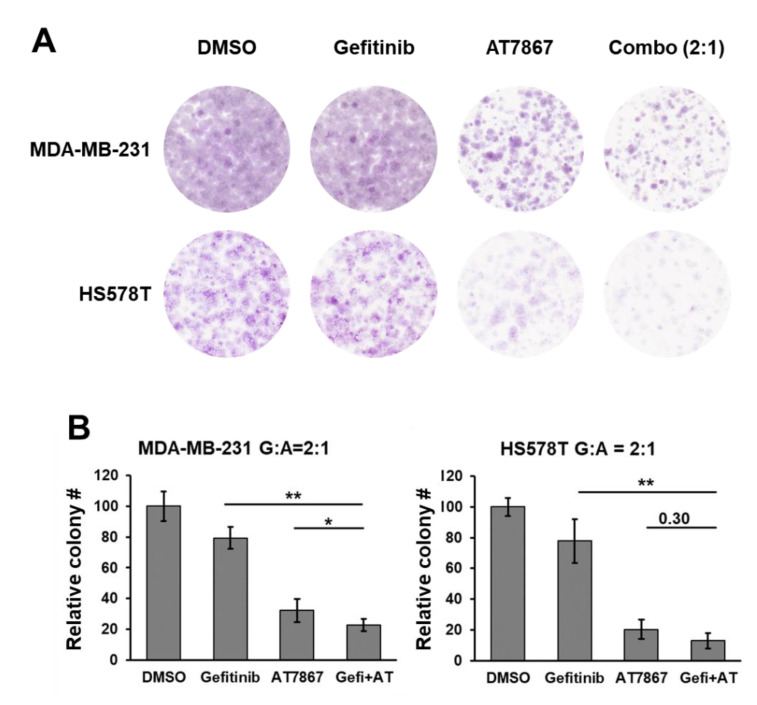
Gefitinib and AT7867 reduced the survival of MSL subtype TNBC cells. (**A**) MDA-MB-231 and HS578T cells were treated with 10 μM Gefitinib, 5 μM AT7867, or 10 μM Gefitinib + 5 μM AT7867 (Gefi+AT) for 24 h in normal growth media. Then, cells were washed and cultivated for additional 10–14 days in normal growth media. The colonies were stained as described in the Materials and Methods. Representative images are shown from three independent experiments performed in triplicate (**B**) The relative colony number was determined and presented as mean ± SEM from three independent experiments performed in triplicate. * *p* < 0.05 and ** *p* < 0.01.

The clonogenic long-term survival of cancer cells was determined to analyze the effect of Gefi+AT7867 treatment. MDA-MB-231 and HS578T cells were subcultured in 6-well plates and treated with drug combinations for 24 h. After washing, the cells were cultivated in normal growth media to form surviving colonies. As expected, gefitinib alone did not suppress the formation of colonies of MDA-MB-231 and HS578T cells (Figure 3A). In contrast to the MTT assay results, AT7867 alone markedly reduced the formation of colonies in both cell types, leading to <40% colony formation when compared with vehicle-treated control cells (Figure 3B). Gefi+AT7867 treatment further reduced the number of colonies in both cells, reaching a statistically significant difference compared with AT7867 alone in MDA-MB-231 cells, but not in HS578T cells. Unfortunately, the complete abolition of colony formation was not achieved by Gefi+AT7867 in either cell type under this condition.

### 3.4. Regulation of Signaling Pathways in TNBC Cells by Gefitinib and AT7867 Treatment

**Figure 4:**
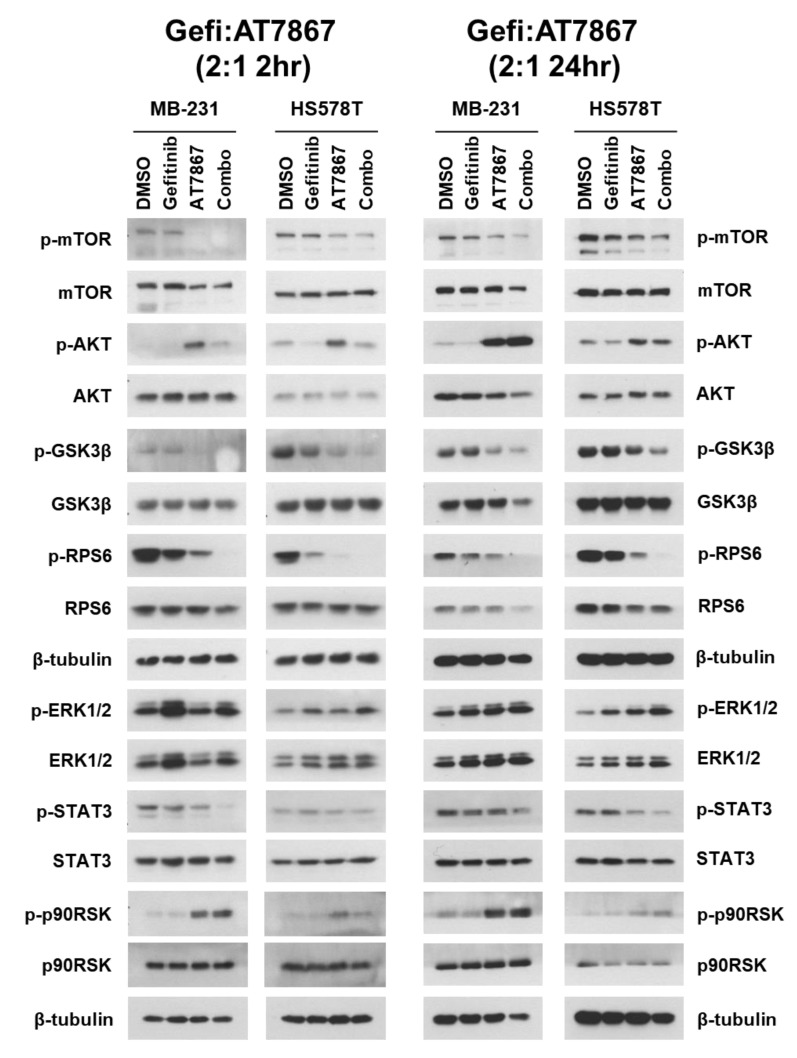
Cell signaling in Gefi+AT7867-treated TNBC cells. Cells were treated with different drug combinations for indicated times. Cell lysates were subjected to Western blot (Figure S1) analysis with antibodies for indicated proteins.

Next, we analyzed the signaling pathways modulated by the Gefi+AT7867 treatment in TNBC cells. As expected, a 2 h treatment of gefitinib was not sufficient to downregulate the AKT pathway, as evident by no significant reduction in the levels of phospho (p)-AKT [25], p-mTOR, and p-GSK3β in MDA-MB-231 and HS578T cells (Figure 4, left). However, the levels of p-RPS6 were reduced by gefitinib alone. Serine 235/236 residues of RPS6 are phosphorylated by multiple protein kinases, including S6 kinase (S6K), ribosomal S6 kinase (RSK), casein kinase 1 (CK1), and protein kinase A (PKA) [109]. The key signaling pathways of p-RPS6 (S235/236) are PI3K/AKT/mTORC1/S6K and RAS/RAF/MEK/ERK/RSK [109]; therefore, gefitinib-induced downregulation of p-RPS6 might be mediated by the RAS/RAF/MEK/ERK/RSK pathway. However, the levels of p-ERK1/2 were increased by gefitinib treatment in MDA-MD-231 and HS578T cells. Additionally, gefitinib did not suppress the levels of p-STAT3 and p-p90RSK in these cells after a 2 h treatment.

AT7867 reduced the levels of p-mTOR, p-GSK3β, and p-RPS6 in these cells after 2 h of treatment. This suggests downregulation of the PI3K/AKT/mTOR/S6K pathway by AT7867. There were no significant effects on the levels of p-ERK1/2 and p-STAT3. Taken together, these results support the specificity of AT7867 and the anti-proliferative and anti-clonogenic effects of AT7867 on MSL TNBC cells (Figure 1 and Figure 2). Surprisingly, the levels of p-AKT and p-p90RSK were upregulated by AT7867 in the cells treated for 2 h. The reactivation of the PI3K or ERK pathway through the relief of the negative feedback loops in cancer cell lines, when treated with PI3K and/or mTOR inhibitors, has been well established [110,111,112,113]. A similar increase in p-AKT (S473) was reported in the glioblastoma cell line U87MG [43].

The Gefi+AT7867 treatment induced further changes in these cells, including the suppression of p-AKT (which was induced by AT7867), the near-complete suppression of p-GSK3β and p-RPS6, and the suppression of p-STAT3 (Figure 4, left). Most of these changes, except for the recurrence of p-AKT, were sustained after 24 h of treatment (Figure 4, right). In addition, the levels of p-ERK1/2 were increased. The levels of p-RPS6, a major regulator of protein synthesis during cell cycle progression, were further reduced by 24 h of Gefi+AT7867 treatment; however, the reactivation of p-AKT and p-p90RSK, and the unregulated maintenance of p-ERK1/2, may contribute to the residual survival of TNBC cells under these conditions.

### 3.5. Upregulation of the MEK/ERK Pathway by Gefitinib and AT7867

Gefi+AT7867 enhanced the anti-proliferative and anti-clonogenic effects in MSL subtype TNBC cells; however, we hypothesized that additional signaling pathways may contribute to the residual survival of TNBC cells in the presence of Gefi+AT7867. For example, the levels of p-AKT were elevated in cells after a 2 h Gefi+AT7867 treatment, and were sustained to 24 h (Figure 4). In addition, p-p90RSK, a downstream target of the RAS/RAF/MEK/ERK pathway, was upregulated by AT7867 and Gefi+AT7867. No significant downregulation was observed in the levels of p-ERK1/2. Interestingly, the levels of p-ERK1/2 were slightly increased by Gefi+AT7867 in cells treated for 24 h (Figure 4, right).

**Figure 5:**
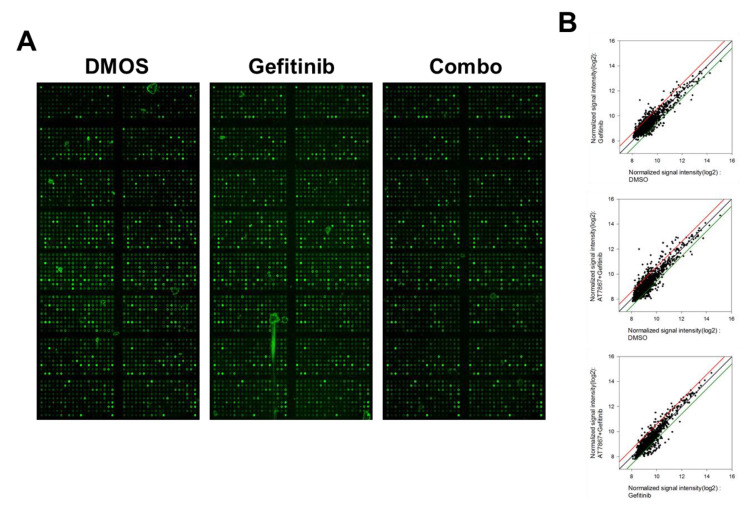

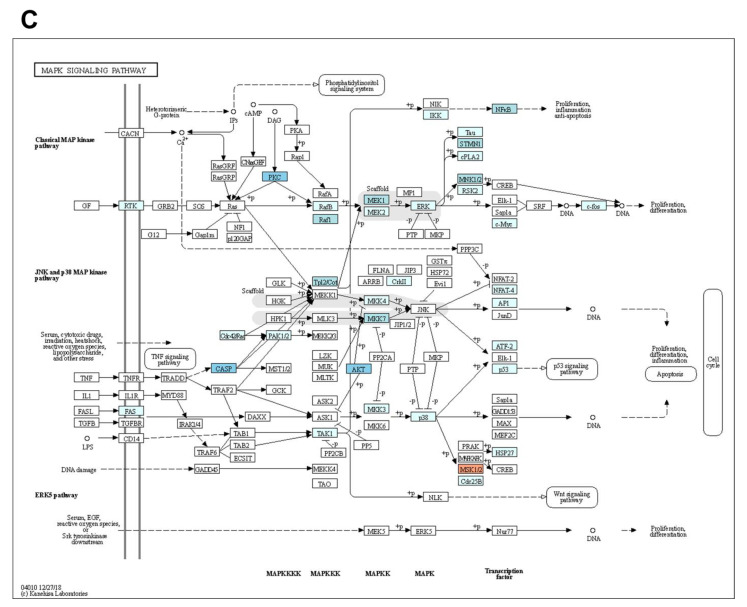
Antibody array analysis. (**A**) Fluorescence images of the antibody arrays. (**B**) Scatter plots of fluorescence signals; intensities of the control sample were plotted on the x-axis, and intensities of the test sample were plotted on the y-axis. Red and green lines indicate a 1.5-fold increase and decrease in fluorescence intensities, respectively, compared with the control sample. (**C**) The Kyoto Encyclopedia of Genes and Genomes (KEGG) pathway map for MAPK/ERK kinase (MEK)/extracellular signal-regulated kinase (ERK) pathway identified by antibody array analysis. Blue color indicates upregulated molecules. The KEGG pathway map was kindly provided by Kanehisa Laboratories (Kyoto University, Kyoto, Japan).

To decipher the pathways that were upregulated in MSL type TNBC cells by Gefi+AT7867, we conducted an antibody microarray analysis. A proteomic expression analysis, comparing gefitinib alone and Gefi+AT7867, was further performed in the Kyoto Encyclopedia of Genes and Genomes (KEGG) pathways database. As a result, we identified that the MEK/ERK pathway was activated by Gefi+AT7867 when compared to gefitinib alone (Figure 5).

### 3.6. Synergistic Enhancement of the Anti-Proliferative Effect of Gefitinib and AT7867 via the Inhibition of the MEK Pathway

The antibody array analysis indicated activation of the MEK/ERK pathway by Gefi+AT7867; therefore, we performed a MTT screening with five MEK/ERK inhibitors (Table 1) in the presence of Gefi+AT7867 in MDA-MB-231 cells. These inhibitors included: (1) CI-1040 (PD184352), an ATP non-competitive MEK1/2 inhibitor (MEK1/2i) with an IC_50_ of 17 nM in cell-based assays [55]; (2) U0126, a selective MEK1/2i with an IC_50_ of 70 nM and 60 nM in biochemical assays [96]; (3) BIX 02189, a potent MEK5 inhibitor with an IC_50_ of 1.5 nM, which also inhibits ERK5 (IC_50_ = 59 nM) in biochemical assays and does not inhibit MEK1/2, ERK2, and c-Jun N-terminal kinase 2 (JNK2) [49]; (4) FR 180204, an ATP-competitive ERK inhibitor with an inhibitory constant (Ki) of 0.31 and 0.14 μM for ERK1 and ERK2, respectively, in cell-free assays [61]; and (5) PD-0325901 (Mirdametinib), a selective non-ATP-competitive MEK1/2i (IC_50_ = 0.33 nM in cell-free assays) [75].

**Figure 6:**
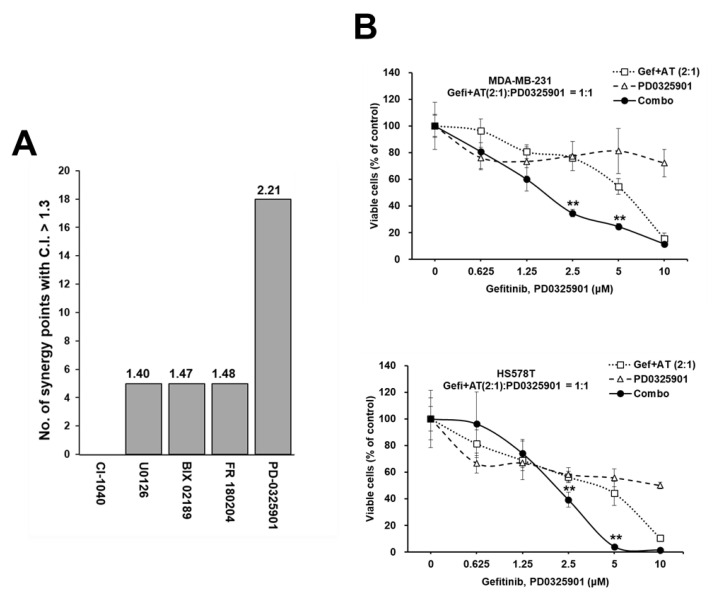
Synergistic anti-proliferative effect in TNBC cells via additional inhibition of the MEK/ERK pathway in the presence of Gefi+A7867. (**A**) Results of the MEK/ERK inhibitor screening with Gefi+AT7867 in MDA-MB-231 cells. The numbers of combination points with a CI > 1.3 are depicted, and the numbers on the top of each bar are the mean CI values of the indicated drugs. (**B**) The combination effects of PD-0325901 in the presence of Gefi+AT7867 in two TNBC cells. Cells were treated with serially diluted concentrations of PD-0325901 in the presence of Gefi+AT7867 for 72 h. Data represent mean ± SD from at least three independent experiments performed in triplicate. ** *p* < 0.01.

As shown in Figure 6A, PD-0325901 was the most potent MEKi to induce strong synergism with Gefi+AT7867 in MDA-MB-231 cells. Furthermore, the triple combination of gefitinib and AT7867 and PD-0325901 reduced MDA-MB-231 and HS578T cell viability (Figure 6B).

### 3.7. Induction of Cell Death by Gefitinib and AT7867 and PD-0325901

**Figure 7:**
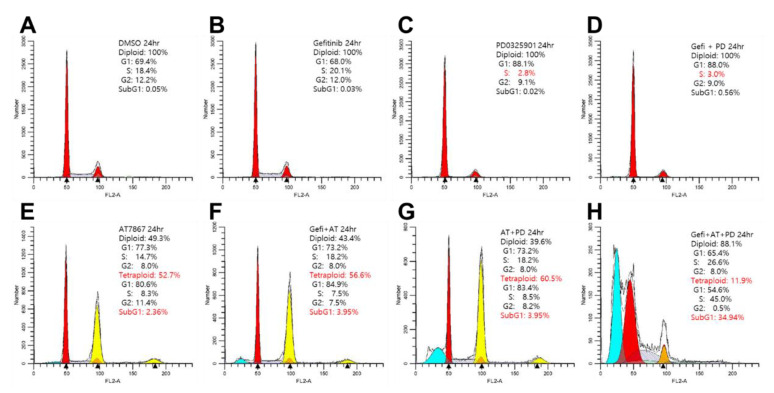
Cell cycle analysis of MDA-MB-231 cells treated with a triple combination of gefitinib and AT7867 and PD-0326901(**A**–**H**). Representative histograms of cell cycle analysis in MDA-MB-231 cells in the presence of each drug or combinations of drugs as indicated. Abbreviations: AT, AT7867; Gefi, gefitinib; and PD-0325901.

Treatment with Gefi+AT7867 for 72 h induced G1 arrest, but not cell death, in MDA-MB-231 cells (Figure 2). Therefore, cell cycle distribution was analyzed in MDA-MB-231 cells treated with the triple drug combination for 24 h (Figure 7). Treatment with PD-0325901 alone increased and reduced the G1 and S phase, respectively, when compared with the DMSO control (Figure 7C). There was no significant increase in the G1 phase with gefitinib (Figure 7B) or AT7867 (Figure 7E) treatment alone; however, the effects of PD-0325901 were sustained in the presence of gefitinib (Figure 7D). By contrast, the effects of PD-0325901 were limited in the presence of AT7867 (Figure 7G). However, the addition of PD-0325901 in the presence of Gefi+AT7867 produced a marked increase in the sub-G1 population (34.94%; Figure 7H). This increase is the result of an increase in apoptotic cells containing fractional DNA content [114,115,116,117,118,119]. Taken together, PD-0325901 induced apoptosis in the presence of Gefi+AT7867 after 24 h, while no significant apoptosis was observed in the absence of PD-0325901 after 72 h treatment (Figure 2).

### 3.8. Blocking Long-Term Survival of TNBC Cells Using Gefitinib and AT7867 and PD-0326901

**Figure 8:**
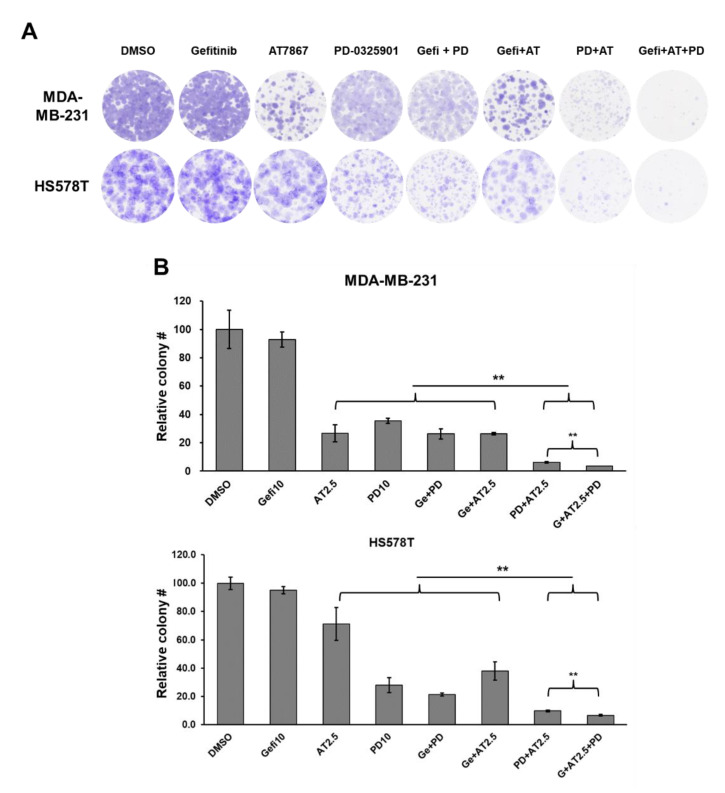
Reduced colony formation of MSL TNBC cells treated with gefitinib andAT7867 and PD-0326901. (**A**) Representative images of the colonies; cells were treated with 10 μM of gefitinib, 2.5 μM of AT7867, and 10 μM of PD-0325901, or other drug combinations (as indicated) for 24 h and further cultivated for 10–14 days to form colonies in normal growth media. The surviving colonies were stained and scanned using an image scanner. Three independent experiments were performed in triplicate and representative images are shown. (**B**) Relative colony numbers were determined and presented as mean ± SEM. ** *p* < 0.01.

The inhibition of the MEK/ERK pathway by PD-0325901 in MSL TNBC cells was analyzed by long-term colony formation assay. As a single agent, 10 μM of gefitinib had little or no effect on MDA-MB-231 and HS578T colony formation (Figure 8). Treatment with 2.5 μM of AT7867 differentially reduced the number of colonies in MDA-MB-231 and HS578T cells. Interestingly, HS578T cells were relatively more resistant to AT7867 than MDA-MB-231 cells (Figure 8B). PD-0325901 (10 μM) alone markedly reduced the long-term survival of both MDA-MB-231 and HS578T cells; however, no significant synergism was observed following the gefitinib and PD-0325901 treatment. Contrarily, PD-0325901 further potentiated the effects of AT7867 in both cells. Moreover, the addition of PD-0325901 resulted in the near-complete inhibition of colony formation of both MSL type cells in the presence of gefitinib and AT7867 (Figure 8). These results suggest that this triple combination could further reduce the survival of residual cancer cells.

### 3.9. Regulation of Signaling Pathways in TNBC Cells by Gefitinib and AT7867 and PD-0326901

**Figure 9:**
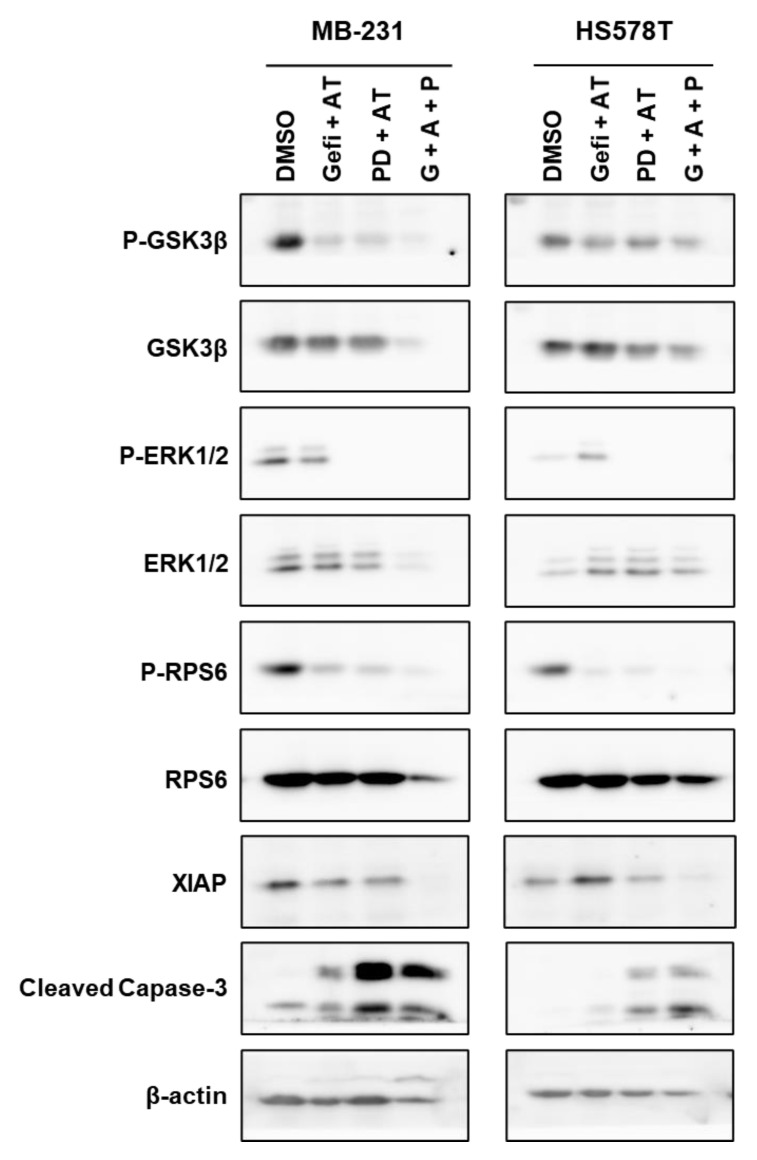
Western blot analysis of TNBC cells treated with different drug combinations (Figure S2). The cells were treated with drug combinations for 24 h, and the cell lysates were resolved on SDS-PAGE and proved with antibodies for indicated proteins.

The effects of this triple combination on the signaling pathways were determined by Western blot analysis. MDA-MB-231 and HS578T cells were treated with drug combinations for 24 h, and the cell lysates were analyzed (Figure 9). As expected, the addition of PD-0325901 abolished the levels of p-ERK1/2 (lanes 3 and 4 in MDA-MB-231 and HS578T). The levels of p-RPS6 were further reduced by PD-0325901 in the presence of Gefi+AT7867. RPS6 was phosphorylated by S6K and RSK [109]; these results support that the inhibition of MEK by PD-0325901 reduces the residual phosphorylation of RPS6 via the MEK/ERK/RSK pathway. Importantly, PD-0325901 induced the cleavage of caspase-3. Inversely, the levels of the X-linked inhibitor of apoptosis protein (XIAP) were reduced by the gefitinib and AT7867 and PD-0326901 treatment. Previously, XIAP was reported as a causative protein in the acquired resistance of TNBC cells to the EGFR/HER2 inhibitor, GW583340 [120]. All these results are consistent with the cell cycle analysis results (Figure 7). Collectively, the addition of PD-0325901 induced the apoptotic cell death of MSL TNBC cells in the presence of Gefi+AT7867.

## 4. Discussion

High levels of EGFR expression in TNBC patients induces activation of the PI3K/AKT, MEK/ERK, and JAK/STAT3 pathways [17,121,122]. The MSL subtype of TNBC is associated with activated EGFR signaling [5] and exhibits greater intrinsic resistance to EGFRis than the BL TNBC cells [25,30]. Inhibition of EGFR alone does not suppress the proliferation of TNBC cells in preclinical cell line models; however, growing evidence supports EGFR as a potential therapeutic target for TNBC treatments, especially in combination with other targeted drugs [3,25,30,33,37,123,124,125,126,127]. Therefore, the identification of targets in combination with EGFRis provides an alternative strategy for developing an effective TNBC therapy because EGFR-targeting drugs have been approved [15,16]. Previously, our group reported that the inhibition of the PI3K/AKT [25], MET [30], or mTORC1 [33] pathways by small-molecule inhibitors induced the sensitization of TNBC cells to EGFRis.

Additional screening with small molecule PKIs in MDA-MB-231 cells identified AT7867 as a combination partner with gefitinib for MSL type TNBC cells. AT7867 is an ATP-competitive inhibitor of AKT1/2/3 (IC_50_ = 32, 17, 47 nM, respectively), p70S6K (IC_50_ = 85 nM), and PKA (IC_50_ = 20 nM) [43]. Interestingly, the PI3K/AKT/mTOR pathway is activated in 10–21% of TNBCs [128]. Here, we showed that AT7867 potentiated the anti-proliferative effect of gefitinib and further reduced the colony formation of TNBC cells in the presence of gefitinib. However, Gefi+AT7867 did not induce apoptotic cell death in TNBC cells. Additionally, Gefi+AT7867 increased the activity of the MEK/ERK pathway. Therefore, we hypothesized that MEK/ERK activation may contribute to the survival of TNBC cells, even in the presence of Gefi+AT7867. In line with this, mutations of Kristen rat sarcoma viral oncogene homolog (KRAS), an upstream regulator of the MEK/ERK pathway, confer EGFRi resistance in lung adenocarcinoma [129].

**Figure 10:**
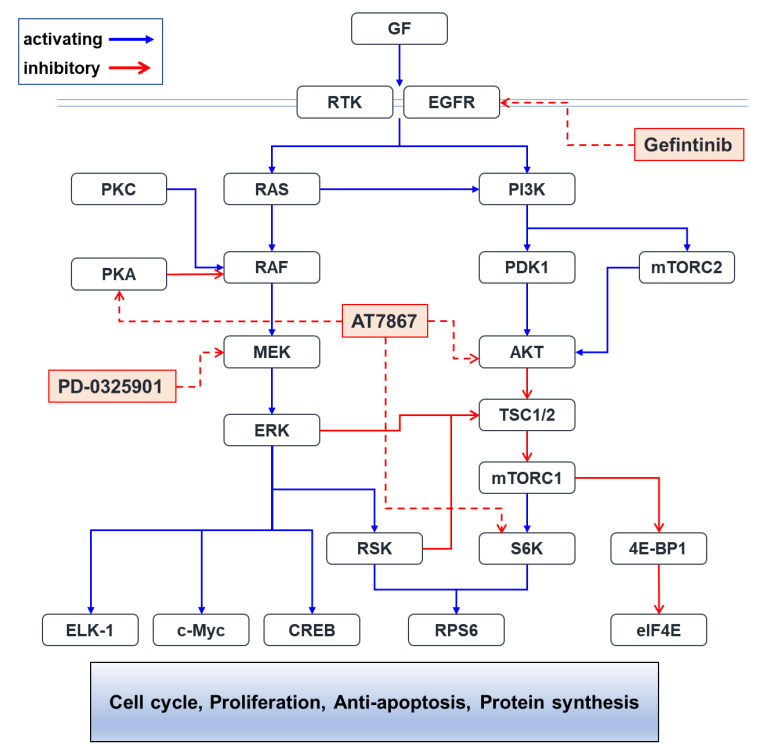
Putative Pathways affected by small-molecule inhibitors in this study. The PI3K/AKT/mTORC1 and RAS/RAF/MEK/ERK pathways cross-talk each other. Small-molecule PKIs used in this study would inhibit their target molecules, which results in synergistic anti-cancer effects. Abbreviations: 4E-BP1, eukaryotic initiation factor 4E-binding protein 1; AKT, v-akt murine thymoma viral oncogene homolog; c-Myc, cellular myelocytomatosis; CREB, cAMP responsive element-binding protein; EGFR, epidermal growth factor receptor; eIF4E, eukaryotic translation initiation factor 4E; ELK-1, E twenty-six (ETS) like-1; ERK, extracellular signal-regulated kinase; GF, growth factor; MEK, MAPK/ERK kinase; mTORC1/2, mammalian target of rapamycin complex 1/2; PDK1, 3-phosphoinositide-dependent protein kinase-1; PI3K, phosphoinositide 3-kinase; PKA, protein kinase A; PKC, protein kinase C; RAF, v-raf-1 murine leukemia viral oncogene homolog; RAS, rat sarcoma; RPS6, ribosomal protein S6; RSK, ribosomal S6 kinase; RTK, receptor tyrosine kinase; S6K, S6 kinase; TSC1/2, Tuberous sclerosis 1/2.

Additional combination screening identified that PD-0325901 (Mirdametinib) induced apoptotic cell death in TNBC cells in the presence of Gefi+AT7867. The inhibition of Gefi+AT7867-induced p-ERK resulted in the induction of cleaved caspase-3 and reduction in the expression of XIAP. Functionally, gefitinib + AT7867 + PD-0325901 markedly diminished the number of surviving colonies. Taken together, attenuating the Gefi+AT7867-induced activation of the MEK/ERK pathway via MEKi overcame resistance to EGFRi (Figure 10). This reduced the survival of residual TNBC cells.

The expression of ERK1/2 is associated with that of EGFR in TNBC tissues [130], and high-level expression of ERK2 is correlated with shorter survival in TNBC patients [131,132]. The RAS/RAF/MEK/ERK pathway is activated by amplification in TNBC, providing potential therapeutic targets [133,134]. Potential anti-proliferative effects of ERK inhibition in TNBC cells have been suggested [135,136,137]. Additionally, kinome analysis has revealed that MEK1/2 inhibition by selumentinib (AZD6244) causes acute loss of ERK activity, leading to the time-dependent reprogramming of receptor tyrosine kinases (RTKs) including platelet-derived growth factor receptor beta (PDGFRβ), discoidin domain receptor tyrosine kinase 1/2 (DDR1/2), and AXL receptor tyrosine kinase (AXL), through ERK-dependent cellular myelocytomatosis (c-Myc) degradation in TNBC cell lines, SUM159 and MDA-MB-231 [138]. These RTK stimulations overcame MEK2 inhibition and reactivated the RAF/MEK2/ERK1/RSK1 pathway, which eventually circumvented MEK inhibition. Dual inhibition strategies have been evaluated for MEKi with other kinase inhibitors to treat TNBC in preclinical models, including EGFRi [32], PI3Ki, PDGFRi [139], and dasatinib [140]. However, phase 2 clinical trials with combination treatments involving the MEKi, trametinib (GSK120212), and the AKTi, uprosertib (GSK2141795), have shown limited efficacy in 50 patients with advanced TNBC [141].

In this report, we found that the triple combination of EGFRi + AKTi + MEKi is more effective at inducing apoptosis and eliminating residual colonies of MSL subtype TNBC cells in vitro. Previous studies suggested that blocking single- or double-signaling pathways was not sufficient to inhibit and induce TNBC cell proliferation and apoptosis, respectively [25,30,33]. This might be because multiple compensatory pathways support the survival of TNBC. In addition, drug-induced reprograming of survival pathways is required to circumvent drug resistance. Further explorations into multiplex drug combinations may provide new insights on treating intractable TNBC.

## 5. Conclusions

In this report, we found that the triple combination of EGFRi + AKTi + MEKi is more effective at inducing apoptosis and eliminating residual colonies of MSL subtype TNBC cells in vitro. Previous studies have suggested that blocking single- or double-signaling pathways is not sufficient to inhibit and induce TNBC cell proliferation and apoptosis, respectively [25,30,33]. This might be because multiple compensatory pathways support the survival of TNBC. In addition, drug-induced reprograming of survival pathways is required to circumvent drug resistance. Further explorations.

## Data Availability

Data in this study will be available from the corresponding authors upon reasonable request.

## Supplementary Materials

The following are available online at https://www.mdpi.com/2072-6694/13/6/1205/s1, Figure S1: uncropped blot images for Figure 4, Figure S2: uncropped blot images for Figure 9.
