# Supplementary material for: Dual Inhibition of AKT and MEK Pathways Potentiates the Anti-Cancer Effect of Gefitinib in Triple-Negative Breast Cancer Cells
Source: Cancers (Basel). 2021 Mar 10;13(6):1205. doi: 10.3390/cancers13061205 (PMC8000364; doi:10.3390/cancers13061205)
Supplement: Supplementary file 1 [file cancers-13-01205-s001.pdf]

# Figure S1

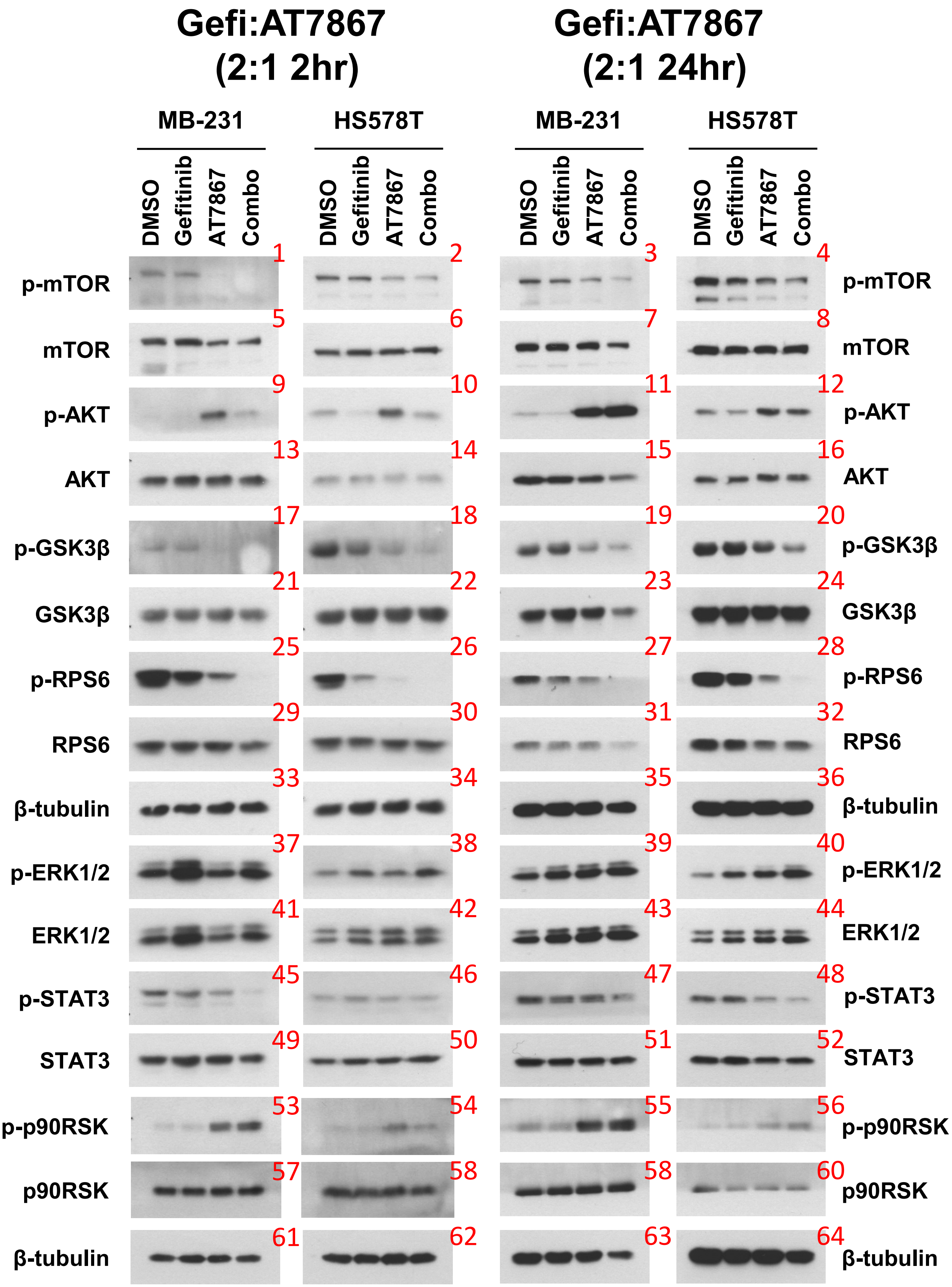

Gefi:AT7867 (2:1 2hr)
Gefi:AT7867 (2:1 24hr)
MB-231
HS578T
MB-231
HS578T
DMSO
Gefitinib
AT7867
Combo
p-mTOR
mTOR
p-AKT
AKT
p-GSK3β
GSK3β
p-RPS6
RPS6
β-tubulin
p-ERK1/2
ERK1/2
p-STAT3
STAT3
p-p90RSK
p90RSK
β-tubulin
1
2
3
4
5
6
7
8
9
10
11
12
13
14
15
16
17
18
19
20
21
22
23
24
25
26
27
28
29
30
31
32
33
34
35
36
37
38
39
40
41
42
43
44
45
46
47
48
49
50
51
52
53
54
55
56
57
58
58
60
61
62
63
64

# Figure S1

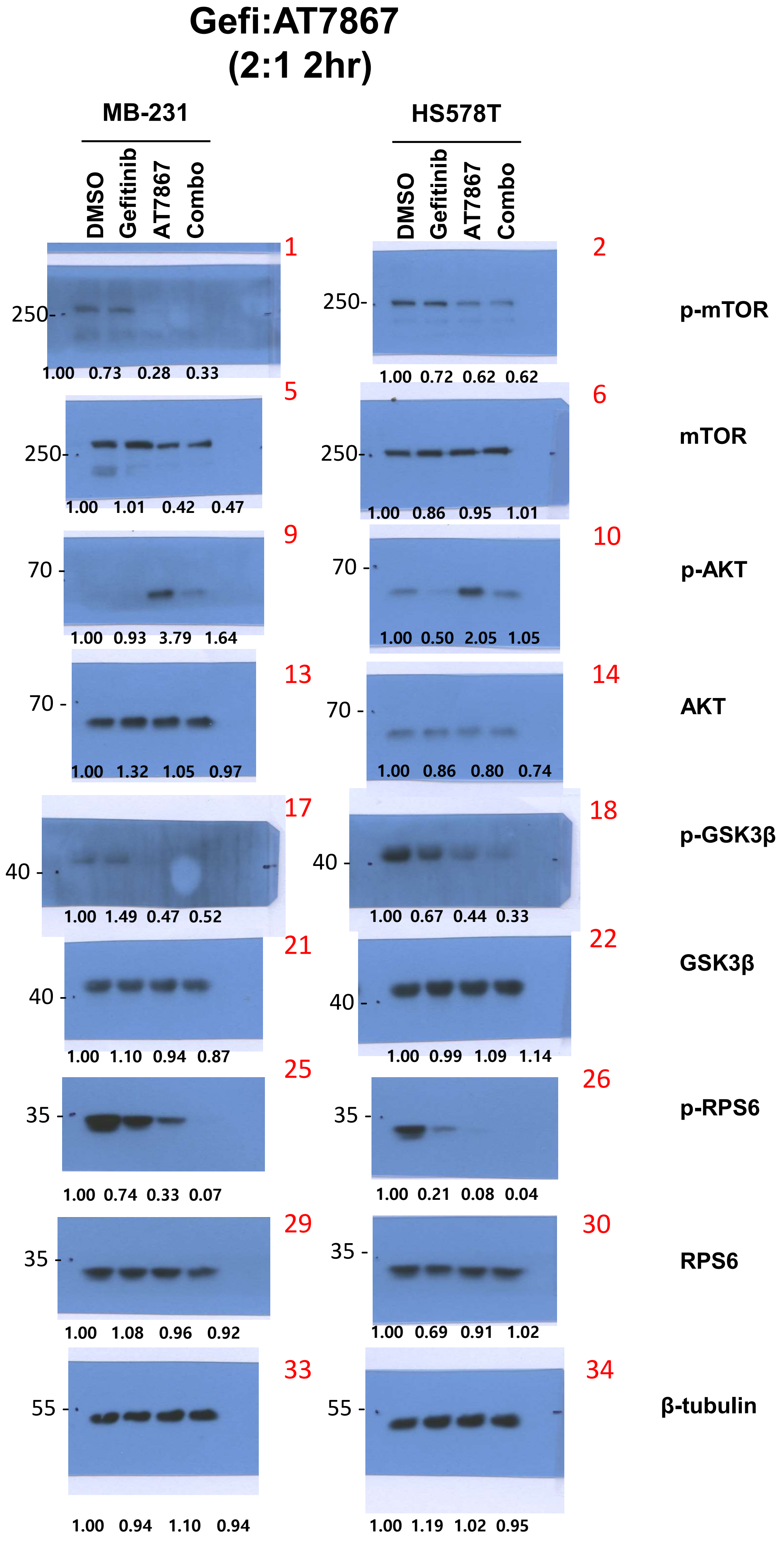

Gefi:AT7867
(2:1 2hr)
MB-231
HS578T
DMSO
Gefitinib
AT7867
Combo
1
2
250-
p-mTOR
1.00 0.73 0.28 0.33
1.00 0.72 0.62 0.62
5
6
mTOR
1.00 1.01 0.42 0.47
1.00 0.86 0.95 1.01
9
10
70 -
p-AKT
1.00 0.93 3.79 1.64
1.00 0.50 2.05 1.05
13
14
AKT
1.00 1.32 1.05 0.97
1.00 0.86 0.80 0.74
17
18
40 -
p-GSK3β
1.00 1.49 0.47 0.52
1.00 0.67 0.44 0.33
21
22
GSK3β
1.00 1.10 0.94 0.87
1.00 0.99 1.09 1.14
25
26
35 -
p-RPS6
1.00 0.74 0.33 0.07
1.00 0.21 0.08 0.04
29
30
RPS6
1.00 1.08 0.96 0.92
1.00 0.69 0.91 1.02
33
34
55 -
β-tubulin
1.00 0.94 1.10 0.94
1.00 1.19 1.02 0.95

# Figure S1

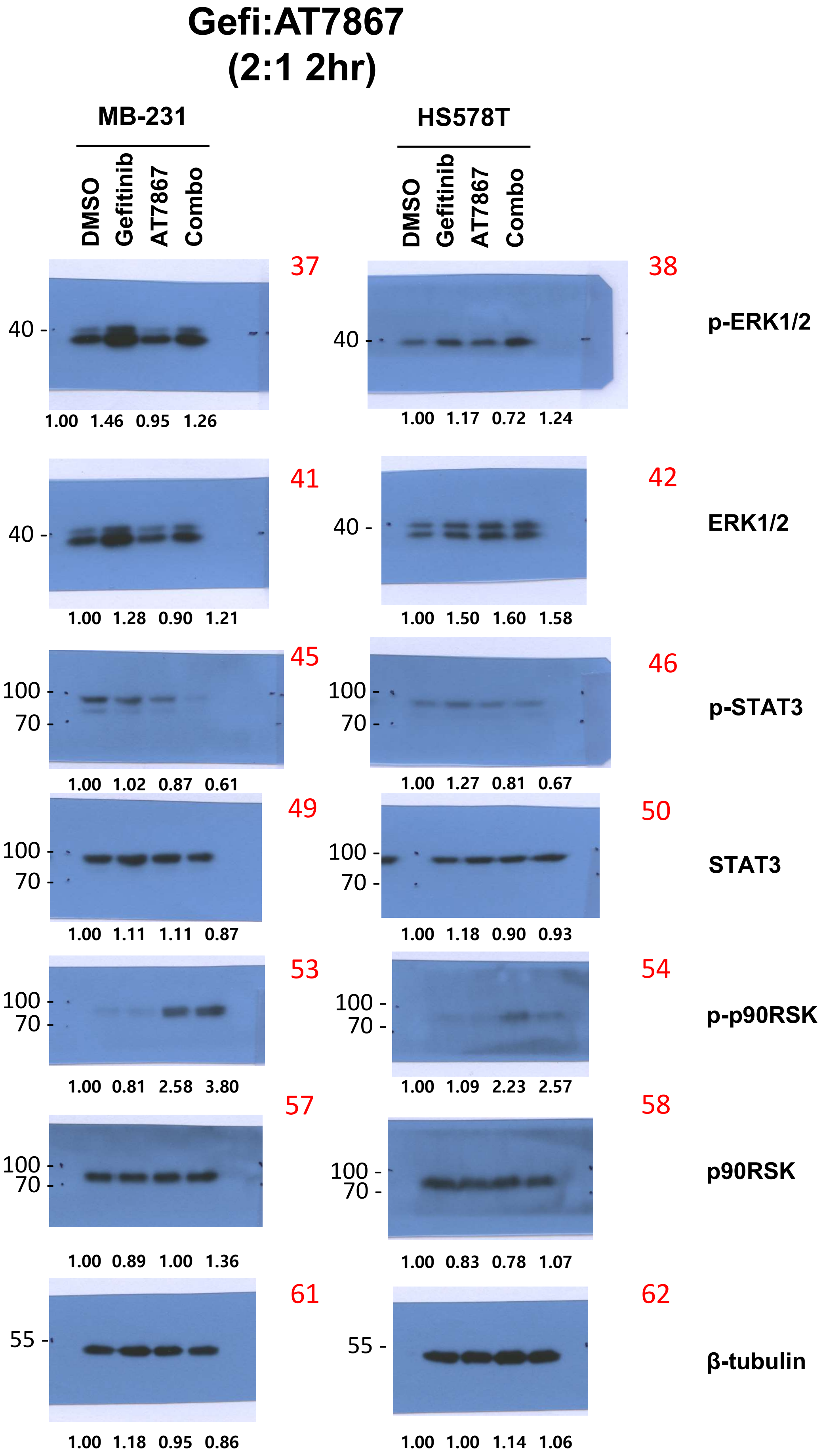

Gefi:AT7867
(2:1 2hr)
MB-231
HS578T
DMSO
Gefitinib
AT7867
Combo
DMSO
Gefitinib
AT7867
Combo
37
38
40 -
40 -
p-ERK1/2
1.00 1.46 0.95 1.26
1.00 1.17 0.72 1.24
41
42
40 -
40 -
ERK1/2
1.00 1.28 0.90 1.21
1.00 1.50 1.60 1.58
45
46
100 -
70 -
100 -
70 -
p-STAT3
1.00 1.02 0.87 0.61
1.00 1.27 0.81 0.67
49
50
100 -
70 -
100 -
70 -
STAT3
1.00 1.11 1.11 0.87
1.00 1.18 0.90 0.93
53
54
100 -
70 -
100 -
70 -
p-p90RSK
1.00 0.81 2.58 3.80
1.00 1.09 2.23 2.57
57
58
100 -
70 -
100 -
70 -
p90RSK
1.00 0.89 1.00 1.36
1.00 0.83 0.78 1.07
61
62
55 -
55 -
β-tubulin
1.00 1.18 0.95 0.86
1.00 1.00 1.14 1.06

**Figure S1**

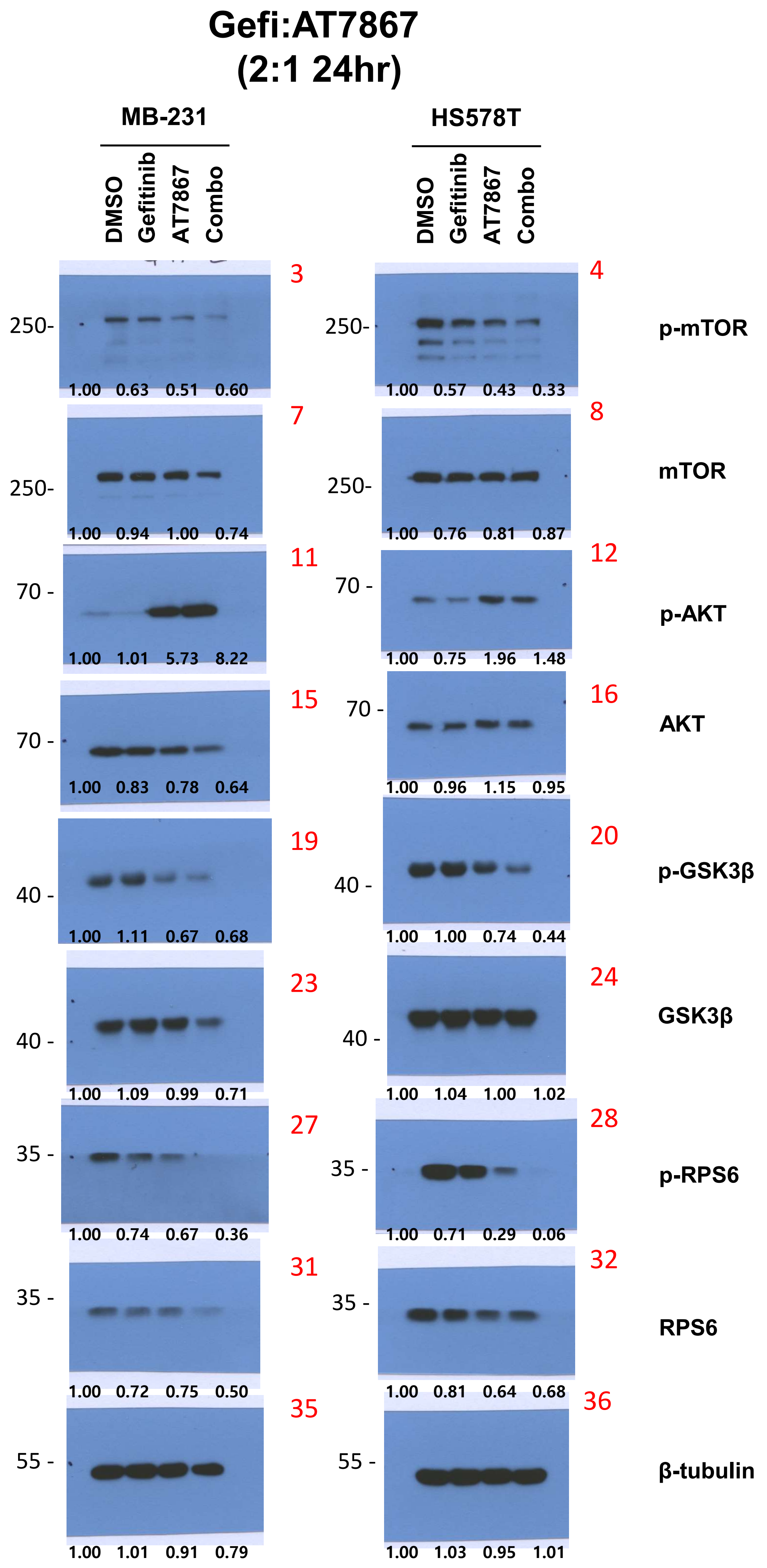
Gefi:AT7867
(2:1 24hr)
MB-231
HS578T
DMSO
Gefitinib
AT7867
Combo
p-mTOR
1.00 0.63 0.51 0.60
1.00 0.57 0.43 0.33
mTOR
1.00 0.94 1.00 0.74
1.00 0.76 0.81 0.87
p-AKT
1.00 1.01 5.73 8.22
1.00 0.75 1.96 1.48
AKT
1.00 0.83 0.78 0.64
1.00 0.96 1.15 0.95
p-GSK3β
1.00 1.11 0.67 0.68
1.00 1.00 0.74 0.44
GSK3β
1.00 1.09 0.99 0.71
1.00 1.04 1.00 1.02
p-RPS6
1.00 0.74 0.67 0.36
1.00 0.71 0.29 0.06
RPS6
1.00 0.72 0.75 0.50
1.00 0.81 0.64 0.68
β-tubulin
1.00 1.01 0.91 0.79
1.00 1.03 0.95 1.01

# Figure S1

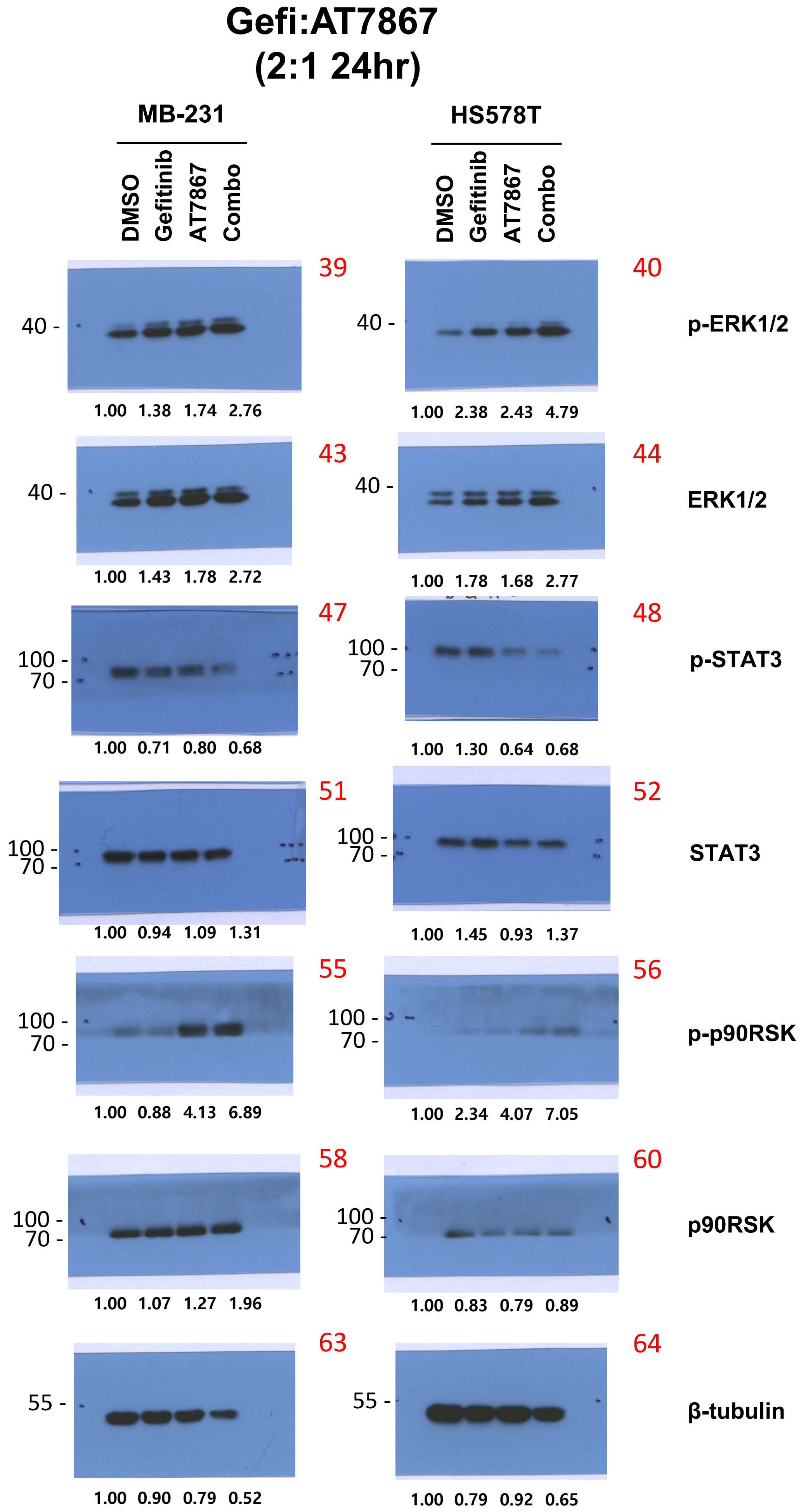
Gefi:AT7867
(2:1 24hr)
MB-231
HS578T
DMSO
Gefitinib
AT7867
Combo
39
40
40 -
p-ERK1/2
1.00 1.38 1.74 2.76
1.00 2.38 2.43 4.79
43
44
ERK1/2
1.00 1.43 1.78 2.72
1.00 1.78 1.68 2.77
47
48
100 -
70 -
p-STAT3
1.00 0.71 0.80 0.68
1.00 1.30 0.64 0.68
51
52
STAT3
1.00 0.94 1.09 1.31
1.00 1.45 0.93 1.37
55
56
p-p90RSK
1.00 0.88 4.13 6.89
1.00 2.34 4.07 7.05
58
60
p90RSK
1.00 1.07 1.27 1.96
1.00 0.83 0.79 0.89
63
64
55 -
β-tubulin
1.00 0.90 0.79 0.52
1.00 0.79 0.92 0.65

# Figure S2

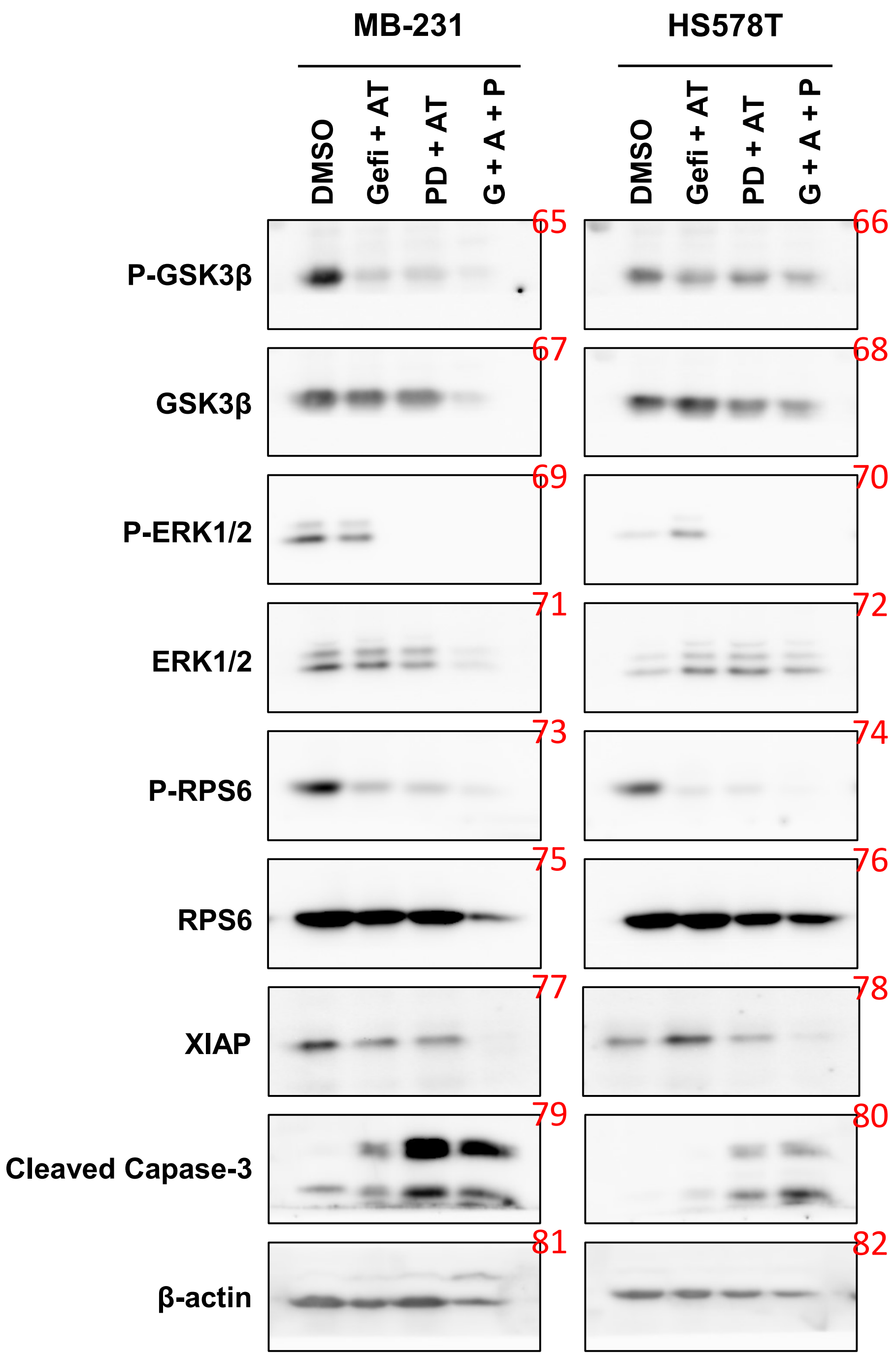

MB-231
HS578T
DMSO
Gefi + AT
PD + AT
G + A + P
DMSO
Gefi + AT
PD + AT
G + A + P
P-GSK3β
GSK3β
P-ERK1/2
ERK1/2
P-RPS6
RPS6
XIAP
Cleaved Capase-3
β-actin
65
66
67
68
69
70
71
72
73
74
75
76
77
78
79
80
81
82

# Figure S2

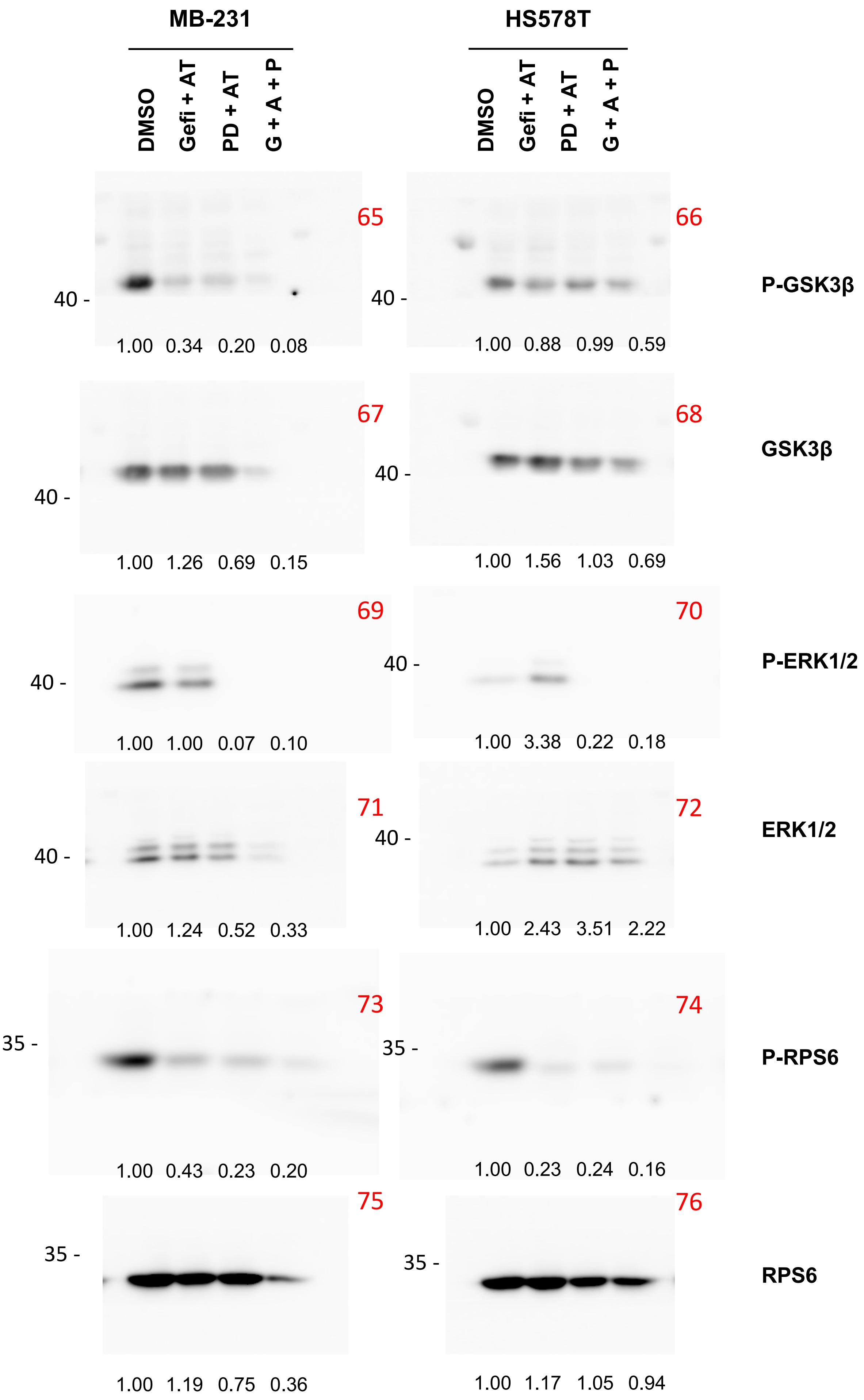
MB-231
HS578T
DMSO
Gefi + AT
PD + AT
G + A + P
65
66
40 -
P-GSK3β
1.00 0.34 0.20 0.08
1.00 0.88 0.99 0.59
67
68
GSK3β
1.00 1.26 0.69 0.15
1.00 1.56 1.03 0.69
69
70
P-ERK1/2
1.00 1.00 0.07 0.10
1.00 3.38 0.22 0.18
71
72
ERK1/2
1.00 1.24 0.52 0.33
1.00 2.43 3.51 2.22
73
74
35 -
P-RPS6
1.00 0.43 0.23 0.20
1.00 0.23 0.24 0.16
75
76
RPS6
1.00 1.19 0.75 0.36
1.00 1.17 1.05 0.94

# Figure S2

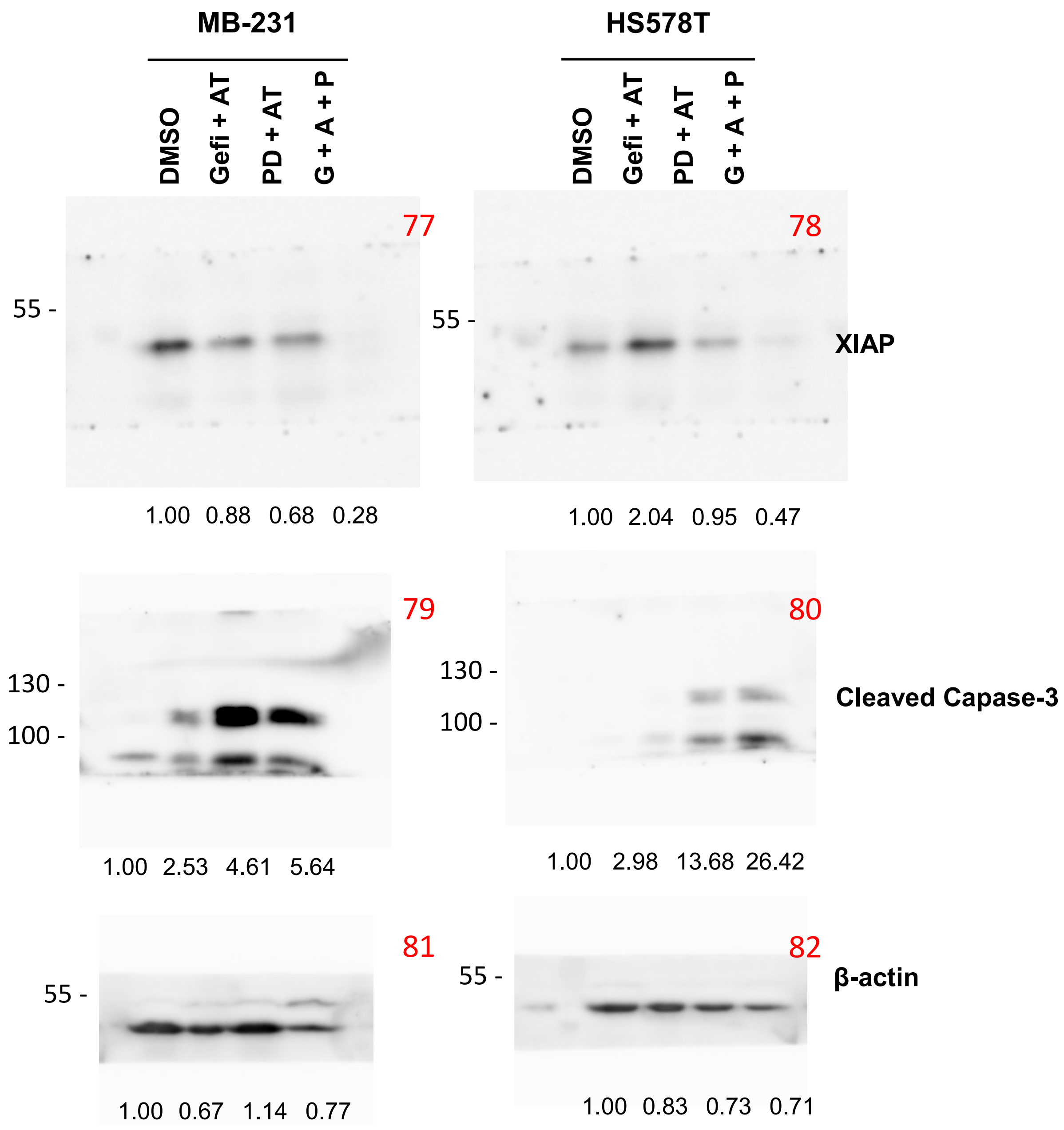

MB-231
HS578T
DMSO
Gefi + AT
PD + AT
G + A + P
77
78
55 -
XIAP
1.00 0.88 0.68 0.28
1.00 2.04 0.95 0.47
79
80
130 -
100 -
Cleaved Capase-3
1.00 2.53 4.61 5.64
1.00 2.98 13.68 26.42
81
82
55 -
β-actin
1.00 0.67 1.14 0.77
1.00 0.83 0.73 0.71
